# Statin therapy reduces oxidized low density lipoprotein level, a risk factor for stroke outcome

**DOI:** 10.1186/cc13695

**Published:** 2014-01-14

**Authors:** Nai-Wen Tsai, Lian-Hui Lee, Chi-Ren Huang, Wen-Neng Chang, Ya-Ting Chang, Yu-Jih Su, Yi-Fang Chiang, Hung-Chen Wang, Ben-Chung Cheng, Wei-Che Lin, Chia-Te Kung, Chih-Min Su, Yu-Jun Lin, Cheng-Hsien Lu

**Affiliations:** Department of Neurology, Chang Gung Memorial Hospital-Kaohsiung Medical Center, Chang Gung University College of Medicine, Kaohsiung, Taiwan; Department of Internal medicine, Chang Gung Memorial Hospital-Kaohsiung Medical Center, Chang Gung University College of Medicine, Kaohsiung, Taiwan; Department of Biological Science, National Sun Yat-Sen University, Kaohsiung, Taiwan; Department of Research, Chang Gung Memorial Hospital-Kaohsiung Medical Center, Chang Gung University College of Medicine, Kaohsiung, Taiwan; Department of Neurosurgery, Chang Gung Memorial Hospital-Kaohsiung Medical Center, Chang Gung University College of Medicine, Kaohsiung, Taiwan; Department of Radiology, Chang Gung Memorial Hospital-Kaohsiung Medical Center, Chang Gung University College of Medicine, Kaohsiung, Taiwan; Department of Emergency Medicine, Chang Gung Memorial Hospital-Kaohsiung Medical Center, Chang Gung University College of Medicine, Kaohsiung, Taiwan

## Abstract

**Introduction:**

Statins are reported to have anti-inflammatory and anti-oxidative effects aside from cholesterol-lowering effects. This study aimed to evaluate the effects of statin therapy on oxidized LDL (Ox-LDL) and the clinical outcome of patients with acute ischemic stroke (AIS).

**Methods:**

This prospective study enrolled 120 patients with AIS divided in the statin (n = 55) and non-statin (n = 65) groups. Eighty sex- and age- matched participants were recruited as risk controls. Ox-LDL was measured using a monoclonal antibody-based enzyme-linked immune-sorbent assay at different time points after AIS. The clinical outcomes were analyzed between the statin and non-statin groups.

**Results:**

Plasma Ox-LDL was significantly higher in stroke patients than in the controls (*P* < 0.001). Plasma Ox-LDL level was significantly reduced in the statin group on day 7 and day 30 compared to the non-statin group (*P* < 0.01). The plasma Ox-LDL positively correlated with serum total cholesterol, LDL-cholesterol, and hemoglobin A1c (HbA1c). Among the potential risk factors, only National Institutes of Health stroke scale (NIHSS) score and Ox-LDL level on admission were independently associated with 3-month outcome.

**Conclusions:**

Our study demonstrates that statin therapy reduces plasma Ox-LDL level after AIS. Plasma Ox-LDL may be a more powerful predictor than serum LDL, high-sensitivity C-reactive protein or white blood cell counts for stroke outcome. Therefore, assay of plasma Ox-LDL should be added as a predictor among the panel of conventional biomarkers in stroke outcome.

## Introduction

Oxidized low density lipoprotein (Ox-LDL) has an established role in the pathogenesis of atherosclerosis. It acts as a pro-inflammatory and pro-atherogenic compound by inducing endothelial dysfunction [1]. In turn, atherothrombosis and oxidative stress play pivotal roles in acute ischemic stroke (AIS) [2, 3]. Previous research has demonstrated elevated levels of circulating Ox-LDL associated with coronary heart disease [4, 5]. Higher concentrations of Ox-LDL are associated with increased incidence of metabolic syndrome [6]. Acute ischemia leads to increased production of free radicals and reactive oxygen species (ROS) in ischemic tissue and plasma [3], and oxidative stress may further oxidize native LDL-cholesterol to Ox-LDL. However, the correlation between circulating Ox-LDL and the clinical outcome of AIS is not well understood.

Statins, the 3-hydroxy 3-methyl-glutaryl coenzyme-A reductase inhibitors, are medications originally employed for the control of hypercholesterolemia [7]. Increasing evidence has demonstrated that statins have pleiotropic effects beyond their lipid-lowering effect [8, 9]. In clinical trials, statin therapy has been shown to reduce cardiovascular events, including myocardial infarction, stroke, and death [10–12]. Although statins are widely used in patients for the reduction of native LDL cholesterol, reports on statin therapy and plasma Ox-LDL are rare, and studies on the effects of statin therapy on Ox-LDL and their association with clinical outcomes of stroke are limited. Thus, this prospective cohort study aimed to test the effects of statin therapy on plasma Ox-LDL, comparing patients receiving and those not receiving statin therapy after AIS. This study also analyzed the predictive value of Ox-LDL on the clinical outcome of patients with AIS.

## Materials and methods

### Study participants

Patients with AIS who were consecutively admitted to the Neurology Department of Kaohsiung Chang Gung Memorial Hospital from October 2012 to April 2013 were evaluated. Acute stroke was defined as acute-onset loss of focal cerebral function persisting for at least 24 hours. The diagnosis of stroke diagnosis was based on clinical presentation, neurologic examination, and results of brain magnetic resonance imaging (MRI) with diffusion-weighted images (DWI). Patients, aged 18 to 85 years, with large-artery atherosclerosis and small artery occlusion were included in the study [13].

For comparison, 80 age- and sex-matched subjects with no clinical evidence of acute cerebral infarction within one year were enrolled as the at-risk control group. Those who received statin treatment before the recruitment were excluded. The Chang Gung Memorial Hospital’s Institutional Review Committee on Human Research approved the study protocol and all of the participants provided informed consent.

### Exclusion criteria

Patients with intracranial hemorrhage were excluded, as well as those with underlying neoplasm, vasculitis, hematologic disorders affecting platelet count or function, end-stage renal disease, liver cirrhosis, or congestive heart failure. Patients with cardioembolic stroke and those who received intravenous thrombolytic therapy were excluded, because those patients undergo a different therapeutic strategy and a high percentage of hemorrhagic transformation. Cardioembolism was diagnosed by clinical presentation, electrocardiography (ECG), and cardiac ultrasound. Patients who had fever, or any infectious disorder within the first week after acute stroke that may affect the Ox-LDL, were excluded. Those who received statin treatment before the index stroke were excluded from the present study.

### Statin treatment and grouping

Patients with AIS were classified into the two groups: the statin group of dyslipidemic patients who started statin therapy after the index stroke event, and the non-statin group of patients who did not receive statin therapy before and after the index stroke. The grouping of patients was dependent on the serum LDL-cholesterol level after stroke. Statin therapy was prescribed among patients with ischemic stroke who had evidence of atherosclerosis and a LDL-cholesterol level ≥100 mg/dL according to the American Heart Association/American Stroke Association guidelines [14].

### Clinical assessments

All of the patients underwent complete neurologic examination upon enrollment and on follow up. Brain MRI with magnetic resonance angiography (MRA), extra-cranial carotid sonography, and transcranial color-coded sonography were performed on patients with ischemic stroke. Detailed medical history was obtained from patients and their families using specific standardized questioning regarding prior use of drugs. Demographic data, history of risk factors, and history of previous vascular events (that is, myocardial infarction, coronary artery disease, and previous stroke) were obtained at baseline. Vascular risk factors included hypertension, or blood pressure >140/90 mmHg on two readings, or currently on anti-hypertensive treatment; diabetes mellitus (DM), or elevated blood glucose on two separate recordings, elevated hemoglobin A1c (HbA1c), or currently on anti-diabetic treatment; and dyslipidemia, or total cholesterol >200 mg/dL, triglycerides >180 mg/dL, or currently on lipid-lowering medication [15].

Neurologic deficits due to stroke were assessed using the National Institutes of Health Stroke Scale (NIHSS). Physical disability and handicap were evaluated using the Barthel index (BI) and a modified Rankin scale (mRS). The NIHSS, BI and mRS were evaluated by investigators (YTC and YFC) blinded to the status of study group on admission and three months post stroke. Functional outcomes were evaluated at three months post stroke. A good outcome was defined as mRS of 0 to 2 without any cardiovascular event, and a poor outcome was defined as mRS of 3 to 6 [16].

#### Measurement of infarct volume

Quantitative measurements of the infarct volume were generated and measured on the DWI. We first selected all images in which the infarct area was displayed as areas of bright signal. On each of these slices, the area of hyper-signal was delineated by an experienced neuroradiologist. Maps of the region of interest used for measurement were stored and then confirmed by a neurologist. The infarct volume was obtained by multiplying the surface by the slice thickness plus the intersection gap.

### Blood sampling and assessment of plasma Ox-LDL

Blood samples were collected by venipuncture of forearm veins from acute stroke patients within 48 hours of the stroke (presented as day 1), and on day 7 and day 30 post stroke. Total cholesterol and triglycerides were measured by enzymatic methods. High-density lipoprotein (HDL) cholesterol was assayed after dextran sulfate magnesium precipitation, and LDL cholesterol was estimated using the Friedewald equation. Plasma Ox-LDL concentration was measured using the mAb-4E6-based enzyme-linked immuno-sorbent assay (Mercodia, Uppsala, Sweden). Briefly, the direct sandwich technique was used wherein two monoclonal antibodies were directed against separate antigenic determinants on the oxidized apolipoprotein B molecule. During incubation, Ox-LDL reacted with anti-oxidized LDL antibodies bound to the micro-titration well. After washing to remove the non-reactive plasma components, a peroxidase-conjugated anti-human apolipoprotein B antibody recognized the Ox-LDL bound to the solid phase. After a second incubation and a simple washing step to remove unbound enzyme-labeled antibody, the bound conjugate was detected through a reaction with 3,3′, 5,5′-tetramethylbenzidine. The reaction was stopped by adding acid to provide a colorimetric endpoint, and was then read using spectrophotometry at 450 nm. Each sample was assayed in duplicate. The intra-assay variation among the duplicates for all samples was <10%. The Ox-LDL concentrations were expressed in U/L.

### Statistical analysis

The quantitative data were presented as mean ± SD. Continuous variables in two groups (that is, controls versus patients, statin group versus non-statin group) were compared using the independent *t*-test for parametric data and the Mann–Whitney *U*-test for non-parametric data. The chi-square test or the Fisher exact test was used for the comparison of proportions between two groups. Repeated measures analysis of variance (ANOVA) was used to compare Ox-LDL at different time points (on days 1, 7 and 30 post stroke), and the Scheffé multiple comparison test was used to analyze the intra-individual course of parameters over time and compare the parameters of two different groups. Pearson correlation was used to analyze associations between Ox-LDL and potential variables.

The independent *t*-test was also used to compare between good- and poor-outcome groups. Multiple logistic regression analyses were used to determine the independent influence of different predictive variables on functional outcome. Statistical significance was set at *P* <0.05. All statistical calculations were performed using the SAS software package, version 9.1 (2002, SAS Statistical Institute, Cary, NC, USA).

## Results

### Demographic data for patients and controls

Of the 160 patients with AIS, 30 were excluded due to statin treatment before the stroke event (n = 17), various infections or fever in the first week after acute stroke (n = 8), cardioembolic stroke (n = 3), and end-stage renal disease (n = 2). The remaining 120 were divided into the statin (n = 55) and non-statin (n = 65) groups. The demographic data for the patients and at-risk controls are shown in Table 1. Age, sex, and other vascular risk factors were similar between the two groups. The white blood cell (WBC) count and serum LDL-cholesterol were significantly higher in the stroke patients than in the controls (*P* <0.01). The plasma Ox-LDL was also significantly higher in the stroke patients (*P* <0.001). There were no significant differences in terms of red blood cell (RBC), platelet counts, HbA1c, serum total cholesterol, HDL-cholesterol, and triglyceride levels.

**Table 1:** **Baseline characteristics and laboratory data for patients with and those without pre-existing statin use on the event of stroke**

	Risk controls (n = 80)	Stroke patients (n = 120)	***P-*** value
Age, y, mean ± SD	58.3 ± 10.7	60.2 ± 10.6	0.08
Sex male, %	70.0	74.2	0.93
Hypertension, %	50.0	72.5	0.12
Diabetes mellitus, %	30.0	35.0	0.86
Hyperlipidemia, %	36.3	47.5	0.12
White blood cells ×10^3^/mL	5.7 ± 2.0	7.6 ± 2.0	<0.001
Red blood cells ×10^6^/mL	4.6 ± 0.4	4.8 ± 0.8	0.36
Platelet counts ×10^4^/mL	20.9 ± 5.8	21.2 ± 6.2	0.77
Total cholesterol, mg/dL	182.1 ± 27.9	192.3 ± 38.8	0.16
LDL-cholesterol, mg/dL	96.4 ± 27.1	119.1 ± 36.2	0.001
Triglyceride, mg/dL	139.3 ± 90.1	141.7 ± 77.7	0.89
HbA1c, %	6.8 ± 1.4	6.9 ± 2.1	0.73
Ox-LDL on admission, U/L	25.9 ± 2.9	33.4 ± 3.0	<0.001

### Laboratory data for the statin and non-statin groups

In the statin group (n = 55), 15 patients used atorvastatin (10 to 20 mg/d), 15 fluvastatin (80 mg/d), 20 rosuvastatin (5 to 10 mg/d), and 5 simvastatin (10 to 40 mg/d). They took the first dose of statin within 72 hours after the onset of stroke. Laboratory data for the statin and non-statin groups are shown in Table 2. Serum total cholesterol, LDL-cholesterol, triglyceride, and HbA1c levels were significantly higher in the statin group than in the non-statin group (*P* <0.001), but the Ox-LDL on admission was not significantly different between the two groups. There were no significant differences in terms of age, sex, vascular risk factors, WBC, RBC, platelet counts, HDL-cholesterol, high-sensitivity C-reactive protein (hs-CRP), blood pressure, NIHSS scores, or BI on admission. There was also no statistical difference in any type of antihypertensive medication between the two groups.

**Table 2:** **Laboratory data for the statin and non-statin groups**

	Statin group (n = 55)	Non-statin group (n = 65)	***P*** -value
Age, y, mean ± SD	61.0 ± 9.4	59.4 ± 11.6	0.40
Sex, male, %	76.4	72.3	0.61
Hypertension, %	69.1	75.4	0.44
Diabetes mellitus, %	36.4	33.8	0.77
Coronary artery disease, %	1.8	10.8	0.07
Intracranial atherosclerosis, %	41.8	30.6	0.21
White blood cells, × 10^3^/mL	7.9 ± 2.0	7.3 ± 1.8	0.13
Red blood cells, × 10^6^/mL	4.8 ± 0.6	4.8 ± 0.9	0.78
Platelet counts, × 10^4^/mL	22.3 ± 5.4	20.4 ± 6.8	0.09
Total cholesterol, mg/dL	214.1 ± 37.5	173.2 ± 26.4	<0.0001
LDL-cholesterol, mg/dL	137.8 ± 37.5	103.0 ± 26.3	<0.0001
HDL-cholesterol, mg/dL	44.5 ± 11.2	44.7 ± 11.7	0.91
Triglyceride, mg/dL	166.5 ± 85.6	120.0 ± 63.2	0.001
HbA1c, %	7.5 ± 2.4	6.5 ± 1.6	0.001
hs-CRP	3.6 ± 0.5	4.04 ± 0.6	0.56
Ox-LDL on admission, U/L	36.3 ± 2.6	34.7 ± 3.0	0.21
Systolic BP, mmHg	145.3 ± 22.4	144.5 ± 28.4	0.87
Diastolic BP, mmHg	83.1 ± 13.6	84.6 ± 13.9	0.57
Median BI (IQR) scores on admission	70 (40 to 95)	65 (45 to 97.5)	0.97
Median NIHSS (IQR) scores on admission	4 (2 to 7)	4 (2 to 8)	0.45
Statin treatment			
Atorvastatin, n	15	-	
fluvastatin, n	15	-	
rosuvastatin, n	20	-	
simvastatin, n	5	-	
With ACEI, n	18	14	0.99
With ARB, n	32	26	0.40
With beta-blocker, n	22	17	0.98
With CCB, n	35	28	0.57

Results are presented as mean ± SD unless stated otherwise. LDL, low density lipoprotein; HDL, high density lipoprotein; HbA1c, hemoglobin A1c; hs-CRP, high-sensitivity C-reactive protein; BP, blood pressure; BI, Barthel index; NIHSS, National Institutes of Health Stroke Scale; ACEI, angiotensin-converting-enzyme inhibitor; ARB, angiotensin receptor blocker; CCB, calcium channel blocker. Dosages: atorvastatin (6 with 40 mg/day and 10 with 10 mg/day); fluvastatin (10 with 80 mg/day); rosuvastatin (14 with 10 mg/day and 2 with 5 mg/day); simvastatin (6 with 40 mg/day and 2 with 20 mg/day).

### Changes in Ox-LDL after AIS in the statin and non-statin groups

**Figure 1:**
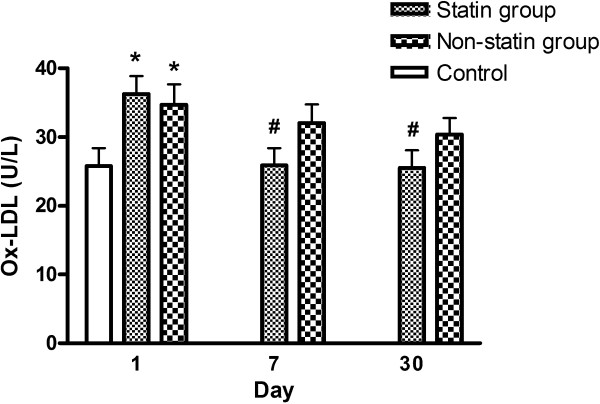
**Changes in oxidized low density lipoprotein (Ox-LDL) level in the statin and non-statin groups after acute ischemic stroke.** *P <0.05 compared to the controls; ^#^ *P* <0.05 compared to the non-statin group.

Changes in plasma Ox-LDL in the statin and non-statin groups are shown in Figure 1. Although the Ox-LDL was similar in the two groups on day 1 post stroke, the Ox-LDL level became significantly lower in the statin group on day 7 and day 30 compared to the non-statin group (*P* <0.01). Repeated ANOVA with the Scheffé multiple comparison test showed significantly different Ox-LDL levels in the two groups at three different time points (on days 1, 7 and 30), even after adjusting for the covariants in terms of total cholesterol, LDL-cholesterol, triglyceride, and HbA1c (*P* <0.05).

### Comparison of Ox-LDL level between diabetic and non-diabetic patients who received statin therapy after AIS

**Figure 2:**
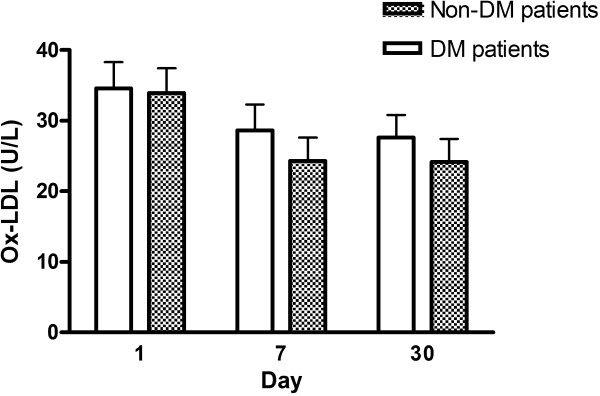
**Comparison of oxidized low density lipoprotein (Ox-LDL) level between the diabetic and non-diabetic patients who received statin therapy after acute ischemic stroke.** DM, diabetes mellitus.

Changes in Ox-LDL level between the diabetic and non-diabetic patients who received statin therapy are shown in Figure 2. Although the Ox-LDL levels showed a reducing trend on day 7 and 30 post stroke when compared to those on admission, there was no statistical difference between the diabetic and non-diabetic patients at any time point (on days 1, 7 and 30).

### Comparison of serial Ox-LDL levels between the good- and poor-outcome groups

To analyze whether the magnitude of change in Ox-LDL levels has any impact on outcome, we used repeated measures ANOVA with the Scheffé multiple comparison test. The result demonstrated that changes in Ox-LDL at three different time points (on days 1, 7 and 30) were significantly different between the two outcome groups (*P* <0.05).

### Correlation between Ox-LDL and potential variables

Pearson parametric correlation between Ox-LDL and potential factors of patients with AIS are shown in Table 3. The Ox-LDL level positively correlated with serum total cholesterol, LDL-cholesterol, and HbA1c levels. Other factors such as age, infarct volume, hs-CRP, WBC, RBC, platelet counts, HDL-cholesterol, and triglyceride did not significantly correlate with plasma Ox-LDL level.

**Table 3:** **Correlation between Ox-LDL and potential variables**

	Ox-LDL	
Pearson correlation	***r***	***P***
Age	0.057	0.540
Total cholesterol	0.476	<0.001*
LDL-cholesterol	0.502	<0.001*
HDL-cholesterol	-0.069	0.470
Triglyceride	0.155	0.097
HbA1c	0.227	0.018**
White blood cells	0.173	0.064
Red blood cells	-0.003	0.978
Platelet counts	-0.062	0.511
hs-CRP	0.030	0.771
Infarct volume	0.059	0.572

*Correlation was significant at 0.001 level (two-tailed); **correlation was significant at 0.05 level (two-tailed). LDL, low density lipoprotein; HDL, high density lipoprotein; HbA1c, haemoglobin A1c; hs-CRP, high-sensitivity C-reactive protein.

### Predictive factors of clinical outcome

The potential prognostic factors of the 120 acute stroke patients for three months are listed in Table 4. Among them, 81 had a good outcome and the remaining 39 had a poor outcome, but no one died during the follow-up period. Statistical analysis revealed that history of coronary artery diseases, NIHSS score, statin therapy, platelet count, serum LDL-cholesterol, hs-CRP and serial Ox-LDL levels were significantly different between the good- and poor-outcome groups. Using the stepwise logistic regression model for these potential variables, only NIHSS score (odds ratio (OR) 1.55, 95% CI 1.20, 1.99; *P* = 0.001) and Ox-LDL on admission (OR 1.09, 95% CI 1.02, 1.18; *P* = 0.009) were independently associated with three-month outcome. Any increase of one U/L in Ox-LDL would worsen the poor outcome rate by 9%.

**Table 4:** **Prognostic factors of patients with acute ischemic stroke**

	Good outcome (n = 81)	Poor outcome (n = 39)	Crude odds ratio (95% CI)	***P-*** value†	Adjusted odds ratio (95% CI)	***P-*** value
Age, y	59.0 ± 10.6	62.6 ± 10.4	1.04 (1.00, 1.08)	0.08	-	-
Sex, male, %	76.5	69.2	0.69 (0.32, 1.48)	0.39	-	-
Hypertension, %	69.1	79.5	1.85 (0.77, 4.43)	0.23	-	-
Diabetes mellitus, %	35.8	33.3	0.86 (0.42, 1.78)	0.79	-	-
Hyperlipidemia, %	48.1	46.2	0.95 (0.47, 1.91)	0.84	-	-
Coronary artery disease, %	2.5	15.4	4.65 (1.29, 16.8)	0.01	-	-
Intracranial atherosclerosis, %	36.3	35.1	1.17 (0.56, 2.45)	0.91	-	-
Patients with Statin therapy, %	54.3	33.3	0.58 (0.29, 1.18)	0.05	-	-
Median NIHSS (IQR) scores on admission	3 (2.0 to 4.5)	8.0 (7.0 to 12.75)	1.43 (1.25, 1.64)	<0.0001	1.55 (1.20, 1.99)	0.001
Laboratory data on admission						
White blood cells, × 10^3^/mL	7.0 ± 2.2	7.6 ± 1.9	0.98 (0.83, 1.16)	0.81	-	-
Red blood cells, × 10^6^/mL	4.9 ± 0.8	4.6 ± 0.6	0.70 (0.41, 1.20)	0.12	-	-
Platelet counts, × 10^4^/mL	22.6 ± 6.2	18.5 ± 5.3	0.99 (0.98, 0.99)	0.001	-	-
Total cholesterol, mg/dL	188.3 ± 35.7	200.6 ± 43.9	1.01 (1.00, 1.02)	0.11	-	-
LDL-cholesterol, mg/dL	114.4 ± 34.2	129.2 ± 38.8	1.01 (1.00, 1.02)	0.04	-	-
HDL-cholesterol, mg/dL	43.8 ± 10.6	46.4 ± 12.9	1.02 (0.99, 1.05)	0.24	-	-
Triglyceride, mg/dL	148.6 ± 79.1	127.2 ± 73.6	0.99 (0.99, 1.01)	0.16	-	-
HbA1c, %	7.0 ± 2.2	6.8 ± 1.9	0.97 (0.82, 1.16)	0.68	-	-
hs-CRP	3.2 ± 0.4	5.34 ± 0.9	1.15 (1.03, 1.30)	0.02	-	-
Systolic BP, mmHg	144.7 ± 24.2	145.1 ± 28.9	1.00 (0.99, 1.02)	0.94	-	-
Diastolic BP, mmHg	83.6 ± 13.6	84.5 ± 14.2	1.01 (0.98, 1.04)	0.75	-	-
Infarct volume, cm^3^	2.5 ± 0.7	4.2 ± 1.1	1.00 (0.99, 1.01)	0.12	-	-
Ox-LDL on admission, U/L†	31.4 ± 2.7	37.7 ± 3.2	1.06 (1.03, 1.10)	0.005	1.09 (1.02, 1.18)	0.009
Ox-LDL on day 7, U/L	28.0 ± 2.7	35.2 ± 23.4	1.07 (1.03, 1.11)	0.008	-	-
Ox-LDL on day 30, U/L	25.6 ± 2.9	32.4 ± 3.2	1.09 (1.03, 1.14)	0.009	-	-

Good outcome: three-month modified Rankin scale (mRS) (0 to 2); poor outcome: three-month mRS (3 to 6).

LDL, low density lipoprotein; HDL, high density lipoprotein; BP, blood pressure; HbA1c, hemoglobin A1c; hs-CRP, high-sensitivity C-reactive protein; NIHSS, National Institutes of Health Stroke Scale; Ox-LDL, oxidized low density lipoprotein. †The odds ratio was calculated by univariate logistic regression.

## Discussion

The present study examined plasma Ox-LDL and the three-month outcome after AIS and had four major findings. First, plasma Ox-LDL was significantly higher in patients with AIS than in at-risk controls. Second, patients receiving statin therapy had lower Ox-LDL levels than those not receiving statin therapy since day 7 after AIS. Third, plasma Ox-LDL level positively correlated with total cholesterol, LDL-cholesterol and HbA1C levels. Last, Ox-LDL level during the acute phase of ischemic stroke was significantly and independently predictive of three-month outcome, and any increase of one U/L would worsen the poor outcome rate by 9%.

The data here are consistent with those of previous studies wherein plasma Ox-LDL was increased in patients with acute cerebral infarction [17, 18]. Previous studies report that circulating Ox-LDL is increased in patients with unstable angina pectoris and acute myocardial infarction, who have atherosclerotic lesions [4, 19, 20]. The mechanism of the rise in plasma Ox-LDL after acute cerebral infarction remains unclear, although it is speculated that AIS is associated with enhanced oxidative stress that can further oxidize native LDL-cholesterol to Ox-LDL [21, 22]. Another possible explanation for the elevation of plasma Ox-LDL may be the increase in lipolysis and lipid peroxidation after cerebral infarction [23, 24]. The longitudinal data here show that plasma Ox-LDL increased in the first few days after stroke onset and gradually decreased to approximate the normal range in the chronic stage. These findings suggest that plasma Ox-LDL may be a useful marker for monitoring the oxidative status of the brain after cerebral infarction.

Although statins are well-known to reduce the native LDL cholesterol, reports related to the effect of statin therapy on the plasma Ox-LDL are rare. To date, this is the first study to demonstrate the association between statin therapy and plasma Ox-LDL in patients with AIS. Our results demonstrate that statin therapy after AIS changed the Ox-LDL level independent of the serum lipid profile and HbA1c. The findings here clarify the role of statin therapy in reducing plasma Ox-LDL effectively in patients after AIS. The results corroborate the efficacy of statins, indicating that lowering LDL means lowering Ox-LDL, especially in patients with AIS, to reduce oxidative stress and endothelial dysfunction [25].

The present study revealed that plasma Ox-LDL is positively correlated to total cholesterol and LDL-cholesterol levels. Because LDL is the substrate for oxidation, concentrations of Ox-LDL correlate with LDL concentrations, and in turn, with total cholesterol. Furthermore, concentrations of Ox-LDL depend on the sensitivity of LDL particles to oxidation, such that the small, dense LDL contains smaller amounts of antioxidants and is therefore more prone to oxidation. Previously, the higher prevalence of small dense LDL has been associated with both AIS onset and short-term mortality after AIS [26]. Unfortunately, the small dense LDL was not assessed in this study. Nonetheless, the results warrant further studies to explore the relationship between plasma Ox-LDL level and the small dense LDL.

In addition, the results show a positive correlation between HbA1C and Ox-LDL that is consistent with findings in previous studies wherein hyperglycemia is associated with increased LDL oxidation, reflective of the rise in Ox-LDL [27, 28]. The association between Ox-LDL and HbA1C may be due to inhibition of insulin signaling by Ox-LDL [29]. A previous study showed that the serum Ox-LDL level increases with the duration of diabetes [30]. The study demonstrated that current diabetes treatment, including statinsm, does not reduce Ox-LDL level in long-standing diabetes. In contrast, our results showed that statin therapy reduced the Ox-LDL level within three months after AIS. The conflict might due to the distinct properties of diseases and different mechanisms of oxidative stress. The acute stroke accelerates oxidative stress in a rapid process, whereas in diabetes mellitus there is continuous chronic oxidative stress.

A novel finding of this study is that Ox-LDL can be used as an easily accessible peripheral marker and as an aid to prognostication in patients with AIS. It can be assumed that the rise in Ox-LDL in patients with AIS may be related to AIS onset via previously mentioned mechanisms. Accordingly, logistic regression analysis (Table 4) reveals that higher plasma Ox-LDL is a predictor for poor three-month outcome after AIS, even after adjustments for other lipid confounders, hs-CRP, WBC, infarct volume and other traditional risk factors for AIS. Although inflammatory biomarkers, such as hs-CRP and neutrophils, have been reported to be useful in predicting clinical outcome after stroke [31, 32], the Ox-LDL seems to to be a better predictor than hs-CRP or WBC counts after AIS in the present study. As yet, it is not possible to conclude whether Ox-LDL is a marker contributing to mechanistic underlying factors on the pathogenesis of cerebral infarction, or whether it is, by itself, a transitional intermediary of this process. However, the association of Ox-LDL with clinical outcome after AIS is consistent with a causal role.

Mounting evidence shows that statins have pleiotrophic effects aside from their cholesterol-lowering effect [8]. Our previous research reported that statin therapy reduces platelet activity and serum hs-CRP level in patients after AIS [12, 33]. In the present study, we further demonstrate that statin therapy reduced the level of Ox-LDL, a risk factor for stroke outcome. Therefore, a further large-scale study is feasible to address the correlation between reduction of Ox-LDL by statin therapy and its impact on stroke outcome.

This study has several limitations. First, this is a prospective cohort study and the grouping of statin therapy is based on the American Heart Association/American Stroke Association guidelines. Thus, it is possible that unmeasured or unknown confounders may have influenced the results. Second, the Ox-LDL level may be influenced by other drugs (for example, anti-platelet, angiotensin II type 1 receptor blockers, and anti-diabetic agents) that may cause potential bias in the statistical analysis [34–36]. Third, the dose and choice of statins was different for each patient, based on the preference of the primary physician. The class or doses of statins that have superior effects have not been analyzed because of the relatively small number of patients in the single center. Fourth, although the sample size was not large, and the follow-up period was short, based on the stepwise analysis, Ox-LDL level on admission remained an important variable predicting outcome. Therefore, the maximum likelihood estimates of the coefficients are valid in the analysis.

## Conclusions

Our study demonstrates that statin therapy reduces plasma Ox-LDL level. The plasma Ox-LDL may be a better predictor than serum LDL, hs-CRP or WBC counts in blood after AIS. Therefore, assay of plasma Ox-LDL should be added as a predictor among the panel of conventional biomarkers in stroke outcome.

## Key messages

- Plasma Ox-LDL was significantly higher in patients with AIS than in at-risk controls.
- Statin therapy reduced plasma Ox-LDL in patients after AIS.
- Plasma Ox-LDL level was a more powerful predictor of outcome than LDL concentration in patients with AIS.

## Abbreviations

ACEI: angiotensin-converting-enzyme inhibitor
AIS: acute ischemic stroke
ANOVA: analysis of variance
ARB: angiotensin receptor blocker
BI: Barthel index
CCB: calcium channel blocker
DM: diabetes mellitus
DWI: diffusion-weighted image
ELISA: enzyme-linked immunosorbent assay
HbA1c: hemoglobin A1c
HDL: high density lipoprotein
hs-CRP: high-sensitivity C-reactive protein
LDL: low density lipoprotein
MRA: magnetic resonance angiography
MRI: magnetic resonance imaging
mRS: modified Rankin scale
NIHSS: National Institutes of Health Stroke Scale
OR: odds radio
Ox-LDL: oxidized low density lipoprotein
RBC: red blood cell
WBC: white blood cell.
